# Supplementary material for: GhRabA4c coordinates cell elongation via regulating actin filament–dependent vesicle transport
Source: Life Sci Alliance. 2022 Sep 6;5(10):e202201450. doi: 10.26508/lsa.202201450 (PMC9449706; doi:10.26508/lsa.202201450)
Supplement: Supplementary file 1 [file LSA-2022-01450_TableS1.docx]

**Table S1 Comparison of fiber quality traits between obtained *GhRabA4c* transgenic lines and the wild type**

| **Accessions** | **Fiber length**  **(mm)** | **Fiber strength (cN•tex^-1^)** | **Fiber micronaire** |
| --- | --- | --- | --- |
| WT | 27.24 ± 0.09 | 23.00 ± 1.25 | 5.29 ± 0.18 |
| 35S:Sense line 28 | 27.51 ± 0.32 | 25.12 ± 2.49 | 5.25 ± 0.02 |
| 35S:Sense line 347 | 27.50 ± 0.26 | 24.66 ± 1.09 | 5.60 ± 0.08 |
| 35S:Sense line 372 (35S-OE1) | 28.43 ± 0.61^**^ | 25.22 ± 0.67 | 5.49 ± 0.41 |
| 35S:Sense line 303 (35S-OE2) | 29.01 ± 0.62^**^ | 24.11 ± 0.71 | 5.00 ± 0.16 |
| RDL:Antisense line 98 (SE1) | 26.72 ± 0.41^*^ | 24.90 ± 0.09 | 4.73 ± 0.53 |
| RDL:Antisense line 217 (SE2) | 26.30 ± 0.54^*^ | 24.43 ± 0.73 | 5.55 ± 0.12 |
| RDL:Antisense line 111 | 26.03 ± 0.25^**^ | 24.53 ± 0.74 | 5.45 ± 0.39 |
| RDL:sense line 56 | 29.51 ± 0.78^**^ | 24.80 ± 1.28 | 5.25 ± 0.21 |
| RDL:sense line 210 (OE1) | 28.77 ± 0.21^**^ | 24.01 ± 0.70 | 5.20 ± 0.15 |
| RDL:sense line 73 (OE2) | 28.73 ± 0.60^**^ | 26.56 ± 2.39 | 5.39 ± 0.14 |

Values represent mean ± SD of three biological replicates. Student’s *t*-test demonstrated that there were significant differences (* P < 0.05 or ** P < 0.01) between the transgenic lines and the wild type.
